# Social media impact on societal security

**DOI:** 10.3389/fsoc.2025.1508542

**Published:** 2025-03-13

**Authors:** Huda Hassan Alnaqbi, Eman Ahmed Mohamed Ali

**Affiliations:** Department of Sociology, University of Khorfakkan, Sharjah, United Arab Emirates

**Keywords:** cybercrime, social media, societal security, social media impact, social networking

## Abstract

This study explores the impact of social media on societal security by surveying 191 employees from various demographic backgrounds. Utilizing a structured questionnaire, the research highlights significant social, ethical, and security consequences, with 81.80% of participants reporting social impacts, 84.40% identifying ethical and behavioral effects, and 82.80% noting substantial security concerns. Despite the broad effects, no significant differences were observed based on gender, education, or social media platform usage. However, age-related variations emerged, with younger groups (23–30 and 31–40) reporting more profound effects compared to those aged 41–50. Based on these findings, the study recommends awareness campaigns addressing the risks of social media, the establishment of legal frameworks to enhance societal security, and the creation of a technical and legal support unit for victims of online incidents. The research calls for further qualitative investigations into hacking and damage cases to deepen understanding and guide effective policy measures.

## 1 Introduction

In today’s digital age, the widespread growth of the digital media sector and information technology has significantly improved communication flexibility through platforms such as Facebook, Twitter, Instagram, and others. These platforms, driven by Web 2.0 technologies, enable global connectivity, fostering social interaction and community building beyond geographical constraints. Despite their advantages, such as accessibility and interactivity, social media also pose risks like cybercrimes and threats to security, making it crucial to evaluate their impact on societal security in the United Arab Emirates (UAE) (Aweida, 2022). The research problem focuses on how social media simultaneously affects societal security, aiming to identify strategies to mitigate risks while preserving its benefits.

The influence of social media on user behavior is a subject of ongoing debate. Some studies praise its positive role in education, addressing traditional teaching limitations and supporting online learning. For instance, Awaj and Tabari (2016) highlight how social media platforms enhance educational experience by providing access to a wealth of resources and facilitating interactive learning environments. Similarly, Abu Rajouh (2021) discusses the benefits of social media in education, emphasizing its role in overcoming conventional educational barriers.

On the other hand, researchers have explored various positive impacts of social media. Al-Sanea (2022) and Yehia and Bzoh (2021) emphasize its role in boosting political and health awareness among university students, showing how these platforms effectively spread information and mobilize public opinion. Similarly, Mohamed and Ali (2018) highlight social media’s strategic value in marketing, particularly its ability to reach target audiences and promote products. Meloudi and Yahya (2019) expand on this, detailing how businesses use social media for branding and engaging customers.

### 1.1 Research gap

However, social media’s impact is not entirely positive. Aweida (2022) highlights language-related challenges caused by widespread social media use, which can harm communication quality and cultural integrity. Ahmed et al. (2021) address social media addiction, noting its negative effects on mental health and productivity. Similarly, Hussein (2020) examines how social media enables cyberbullying, contributing to harmful behaviors and affecting individual wellbeing.

### 1.2 Objectives

The current study aims to achieve several key objectives:

Highlight the social impacts of social media on societal security in the United Arab Emirates

1. Elucidate the ethical and behavioral effects of social media platforms on societal security.
2. Understand the intellectual security effects of social media on the societal level in the UAE.

### 1.3 Significance

The significance of the study lies in enriching scientific literature with its results and information, which may prove beneficial for those interested in the fields of societal security and social communication. The study also sheds light on prominent points and influential as experiences and media sites and applications, emphasizing their role as a vital means for exchanging ideas and experiences, and underscores their importance in enhancing communication among individuals and groups. By highlighting the risks of certain practices and content shared through social media, the study contributes to raising awareness among different societal segments about the importance of family and community oversight. Additionally, the study emphasizes that it serves as a crucial starting point for further future research on this vital topic.

## 2 Theoretical framework

### 2.1 Concept of social media

#### 2.1.1 The concept of social media

Social media, defined diversely, includes electronic networks for communication, responsive communication to alter attitudes, global networks for worldwide connections, virtual sites for global interaction, internet-based platforms for communication, and modern applications enabling easy interaction (Al-Omari, 2018). Social media platforms encompass a broad range of digital communication channels that facilitate the sharing of information, ideas, and multimedia content among users. These platforms enable individuals to create and share user-generated content, engage in real-time interactions, and connect with others across geographical boundaries. From social networking sites like Facebook and Twitter to multimedia-sharing platforms like Instagram and YouTube, social media platforms have become integral to modern communication practices (Qadri, 2016).

#### 2.1.2 Characteristics of social media

Social media stands out with various features that distinguish it from other programs and applications. Some of these features include the ability for users to comment on content, express their opinions, and share various forms of content such as texts, images, and videos. The speed and accuracy in exchanging information and files are notable, and users can access social media platforms through laptops or smartphones. Additionally, social media serves as an effective and rapid marketing tool, allowing individuals to connect with people worldwide, fostering social relationships, and enabling positive change through the exchange of experiences and knowledge (Qandilji and Al-Samaraie, 2012).

Social media platforms are defined by their interactive nature, enabling real-time communication and collaboration. Users can share multimedia content like photos, videos, and audio, enriching communication and fostering creativity. These platforms often use algorithms to personalize content based on user preferences, creating a tailored experience. Their widespread availability across devices ensures seamless connectivity and accessibility for users globally.

#### 2.1.3 Importance of social media

Social media platforms, highlighted by Suleiman (2015), play a vital role in communication, relationship building, and influencing social processes. A study by Ellouze and Derouiche (2022) emphasizes social media’s impact on enhancing individual social skills.

The importance of social media surpasses the function of communication to become a strong tool for networking and relationship-building. It connects one with friends, family, colleagues, and similar-minded individuals across the globe. Further, public discourse is influenced, societal attitudes shaped, and social movements driven-from political activism to cultural movements-on these very platforms, making them potent catalysts for awareness and transformation.

## 3 Purpose of social media and its types

Social media platforms serve diverse purposes, including business, social, political, educational, religious, and entertainment goals (Ali, 2014). Various types cater to specific needs, from public networks like Facebook and Twitter for broad discussions to private networks like LinkedIn for professional networking.

### 3.1 Purpose of social media platforms

Social media sites bring together different ideas for various users in many areas. They have been used to communicate, collaborate, share information, and build networks-the reason they have become an important part of modern life. Some sites specialize in one area, such as professional networking and career advancement, as seen with LinkedIn, which helps users highlight skills, connect with industry professionals, and find new job opportunities.

On the other hand, platforms like Facebook and Twitter are popular for their broad reach and engagement, making them ideal for social interactions, sharing news and updates, and fostering online communities. Educational institutions and organizations utilize social media platforms to enhance learning experiences, facilitate knowledge sharing, and engage with students and stakeholders. Moreover, religious communities and organizations leverage social media to disseminate religious teachings, connect with followers, and organize events and gatherings.

### 3.2 Types of social media platforms

Social media typologies are diverse, each specialized in serving different needs and preferences of users. Public networks like Facebook and Twitter allow for wide-based discussions, sharing of updates and news, and connectivity with a vast audience. These have been considered open platforms, accessible to a wide range of users.

Private networks, such as LinkedIn, prioritize professional networking and career development, offering features tailored to the needs of professionals and businesses. Users can create detailed profiles highlighting their skills and experiences, connect with colleagues and industry peers, and explore job opportunities and career resources.

Additionally, platforms like YouTube, Instagram, and WhatsApp cater to specific content sharing needs, with YouTube focusing on video content, Instagram on visual storytelling, and WhatsApp on instant messaging and communication. These platforms offer unique features and functionalities that appeal to different user demographics and preferences.

## 4 Social media platforms properties

### 4.1 General properties

Social media platforms, highlighted by Hatimi (2015), share common traits such as user-generated content, easy communication, and user autonomy in content selection. Hatimi (2015) emphasizes their distinctive features, including spontaneous communication without formalities, cost-effectiveness, and breaking hierarchical barriers.

Social media platforms are user-centric; they allow users to create, share, and interact in real time. User-generated content is the drive behind these networks, which enables users to be creative and connect with others based on topics of interest. Ease of communication engenders spontaneity in interactions and community bonding. Users’ autonomy to choose and consume preferred content makes the experience personalized and customized.

### 4.2 Advantages and disadvantages

Despite the numerous positive aspects, social media comes with both benefits and drawbacks. Positive aspects include global connectivity, easy interaction, versatility for all age groups, user-friendly interfaces, economic accessibility, rapid information exchange, and advertising opportunities for businesses (Ismail, 2019).

Social media platforms provide unmatched opportunities for global connection, transcending geographical and time barriers. Their ease of interaction enables seamless communication and collaboration, promoting cultural exchange and cross-cultural understanding. Additionally, these platforms cater to all age groups, offering intuitive interfaces and features that adapt to diverse preferences and needs.

However, negatives involve risks such as exposure to explicit content, privacy breaches, dissemination of extremist and racist ideas, sharing inappropriate images, spreading vulgar language, impact on family bonds, susceptibility to online scams, and the potential for fostering feelings of frustration and despair due to idealized portrayals on social media (Al-Oraishi and Aldossari, 2015).

While social media pervades our lives, it also raises great concern about abusive content and the consequences on mental and emotional wellbeing. Further, cyberbullying and online harassment come with some risks, thus calling for active action in their handling. Furthermore, personal information is threatened by breaches of privacy and misuse of data, hence highlighting the need for robust controls on privacy and regulatory mechanisms.

## 5 Services and functions of social media

Social media platforms provide diverse services and functions that enable personal and social interactions. Key features include personal profiles, where users share details like names, photos, and interests, fostering connections based on shared interests or goals. While these platforms enhance communication, they also pose challenges such as family disruption, fake relationships, misinformation, and health concerns. Users must balance their engagement to mitigate these risks (Sahari and Dabih, 2017). Additionally, social media serves as a hub for building online identities and expanding social networks through meaningful interactions (Al-Rahamneh, 2018).

However, the widespread use of social media has led to negative consequences, such as disrupted family dynamics, fake relationships, the spread of misinformation, and potential health issues. Users must balance their engagement with these platforms and prioritize their wellbeing, being mindful of the risks linked to excessive usage.

## 6 Misuse of social media and its solutions

Misuse of social media poses security risks at national, societal, and individual levels, threatening stability and cultural identity. Legal updates, international cooperation, and regulatory controls are needed (Ghanem, 2010). Strategies to combat the misuse of social media platforms involve a multi-faceted approach that encompasses legal, regulatory, and awareness-raising measures. Creating a National Cybersecurity Authority and implementing regulatory controls on media are proposed solutions (Bazarqan, 2008). These initiatives aim to enhance oversight and accountability in the digital sphere, ensuring that social media platforms operate in compliance with established laws and regulations.

Raising awareness about the risks associated with social media misuse is crucial for empowering individuals to make informed decisions about their online activities. Recommendations for monitoring, guidance, and training are essential components of effective awareness-raising efforts (Awawdeh, 2020). By educating users about the potential dangers of social media misuse and providing them with the tools and resources to protect themselves online, these initiatives can help mitigate security risks and promote responsible digital citizenship (Eido, 2020).

## 7 Community security

Throughout history, the concept of community security has evolved, emphasizing the importance of unity and stability. In contemporary society, threats to community security, such as the spread of rumors on social media (Al-Sa’eeda, 2019), are recognized. Maintaining community security is essential for progress, prosperity, and preserving social bonds, as rumors can erode relationships and trust (Abdel, 2010).

Community security is the state of tranquility resulting from the absence of conflicts with community values (Al-Osaimi, 2004). It arises from belonging, stability, and meeting basic needs (Jellab, 2020). This definition underscores the importance of social cohesion, stability, and the fulfillment of basic human needs in ensuring community security.

Community security is important in providing the base for a healthy and successful society. It nurtures social cohesion, trust, and cooperation, hence creating an environment that allows individuals to flourish and contribute to the common good. Moreover, it secures against external threats and offers stability in society during challenges and uncertainties.

Community security is vital for comprehensive development, impacting education, health, industry, and agriculture. It attracts foreign investments, ensures project stability, and facilitates regional and international collaboration for environmental protection. Lack of community security leads to conflicts, resource depletion, and hinders environmental efforts. Overall, community security is crucial for wellbeing, stability, and holistic development (Al-Kasasbeh, 2017).

Community security and development share a symbiotic relationship, each reinforcing the other. A secure community lays the foundation for economic growth, social progress, and environmental sustainability. Conversely, development initiatives that improve living standards, promote education, and foster innovation enhance community security by addressing root causes of insecurity and building resilience. Thus, investing in community security is both a moral and strategic necessity for sustainable development and a prosperous future.

## 8 Connection with the UAE society

Research conducted on UAE Facebook users demonstrates that social media primarily serves as a tool for maintaining family connections and following news of relatives and friends, particularly when direct communication is challenging (Aldhmour, 2022). This aligns with the UAE’s strong family-oriented culture, though it simultaneously presents challenges to traditional face-to-face social interactions that have historically characterized Emirati society.

Within the UAE context, social media’s impact on societal security manifests through several channels (Aldhmour, 2022):

1. *Individual security*: UAE residents face potential risks including:

- Vulnerability to cybercrime
- Reduced workplace productivity due to social media addiction
- Deteriorating academic performance among students
- Weakening of real-world social connections

2. *Family security*: The impact on Emirati families includes:

- Introduction of undesirable behaviors and language within family settings
- Challenges to traditional family authority structures
- Widening generational gaps
- Potential weakening of family bonds

3. *Community security*: Effects on the broader UAE community include:

- Reduced participation in traditional community activities
- Potential erosion of social cohesion
- Impact on established customs and traditions
- Introduction of foreign cultural elements that may conflict with local values

## 9 The global perspective

According to research from the University of Oxford, approximately 70 states, including India and various Arab and Western nations, have been actively utilizing and manipulating social media to achieve their foreign and national policy objectives. The study revealed that 90% of National Security Agencies (NSAs) have been manipulating media for multiple purposes, including recruitment, fundraising, networking, training, and influencing. This global trend is exemplified by the 2010 Arab Spring, which many termed the “Facebook Revolution” due to social media’s significant role in amplifying the uprising. While debate continues about whether Facebook was solely responsible for the Arab Spring, its impact on facilitating activist communication and amplifying demands was substantial, though by the time social media platforms recognized the extent of the chaos, many considered their intervention too late (Kasi et al., 2021).

The global response to social media’s security implications has varied significantly across nations. China, for instance, has taken a protective stance by restricting western social media giants and developing its own domestic platforms. This contrasts with countries like Pakistan, where approximately 18% of the population actively uses social media, primarily between ages 18–24, despite limited resources for cybercrime monitoring. The challenge of maintaining national security in the digital age is further complicated by hostile groups utilizing social media for opinion building, propaganda dissemination, and cybercrime activities. This has led to increasing recognition of the need for governments to develop native social media platforms and implement more effective monitoring systems to protect national interests (Kasi et al., 2021).

## 10 Societal security

Societal security manifests through state institutions’ capacity to combat and limit crime proliferation (El-Derouby, 2018). The decline in crime rates serves as a key indicator of societal security, while increased rates signify its absence. State institutions strive to enforce order and uphold the rule of law through criminal justice systems, sometimes necessitating force against lawbreakers and those who seek to disrupt social order, thereby enhancing feelings of justice and reducing crime-related fear while building trust in state institutions (Kasemy and Geday, 2019).

Security represents one of society’s fundamental needs and serves as an indicator of societal progress, prosperity, and stability (Bny Saleh, 2021). Communities with strong security frameworks demonstrate enhanced development and achievement, as security facilitates work, creativity, stability, and productivity while preserving national identity. Security stands as a cornerstone of stability, protecting individuals and groups from internal and external threats, particularly various forms of crime within society (El-Agha, 2009). Societies plagued by widespread crime lack security, potentially leading to their deterioration, underdevelopment, and instability.

Previous studies

1. Jain et al. (2021) this study provides a comprehensive review and analysis of security and privacy issues associated with online social networks (OSNs). By examining various threats and existing solutions, the paper offers valuable insights into safeguarding user information and enhancing trustworthiness in online social networks. Our study benefits from this research by building upon the foundational knowledge presented in this paper and expanding our understanding of the security and privacy challenges posed by social media platforms.
2. Olan et al. (2024) this study investigates the impact of fake news (FN) on social media (SM) and its ramifications on societal values and perceptions. By proposing a conceptual framework and conducting survey analysis, the research sheds light on the divisive nature of fake news and its potential to reshape societal beliefs and norms. Our study benefits from this research by considering the broader societal implications of social media usage, particularly in relation to misinformation and its influence on societal security.
3. Rao and Kalyani (2022) this study explores the positive and negative effects of social media on society, providing insights into its role in shaping interpersonal relationships, professional networking, and societal behaviors. By examining both the benefits and risks associated with social media usage, the paper offers a nuanced understanding of its impact on various aspects of society. Our study benefits from this research by considering the multifaceted nature of social media’s influence and its implications for societal security and wellbeing.
4. Zhang and Gupta (2018) this study investigates the security and trustworthiness issues inherent in social media platforms, proposing a novel approach for evaluating and measuring their effectiveness in addressing emerging threats. By highlighting the need for enhanced security measures and trust-building mechanisms, the research offers valuable insights into mitigating risks and enhancing user confidence in social media environments. Our study benefits from this research by considering the broader implications of social media security and trustworthiness for societal security and resilience.
5. Olan et al. (2024) this study investigates the impact of fake news (FN) on social media (SM) and its ramifications on societal values and perceptions. By proposing a conceptual framework and conducting survey analysis, the research sheds light on the divisive nature of fake news and its potential to reshape societal beliefs and norms. Our study benefits from this research by considering the broader societal implications of social media usage, particularly in relation to misinformation and its influence on societal security.

## 11 Materials and methods

The study employed a descriptive-analytical methodology, which was selected for several key reasons. First, this approach enables the systematic collection and analysis of comprehensive data about complex social phenomena, particularly suited for examining the multifaceted impacts of social media on societal security (Darwish, 2018, p. 118). Second, the descriptive-analytical method allows for both quantitative measurement of observed effects and qualitative interpretation of patterns and relationships, making it ideal for investigating the various demographic variables involved in this study.

The study population comprised all employees of the University of Khorfakkan, United Arab Emirates, as of 2022, totaling 400 male and female employees. This population was chosen because it represents a diverse cross-section of working professionals with varying levels of social media engagement and different demographic characteristics, making it suitable for examining societal security concerns across multiple variables.

A simple random sampling method was employed to ensure unbiased representation and statistical validity. The sample size was determined using Steven Thompson’s equation, which is particularly appropriate for finite populations and provides a statistically robust sample while maintaining practical feasibility. This equation yielded a target sample size of 196 individuals. To enhance the reliability of the research instrument, a pilot study was conducted with 30 randomly selected participants, whose responses were subsequently excluded from the final analysis. The final study achieved a high response rate of 97.45% (191 employees), indicating strong engagement and representativeness of the sample (Darwish, 2018).

### 11.1 Alignment of the methodology to the research objectives

The descriptive-analytical methodology was specifically chosen to align with the study’s objectives of understanding the multifaceted impacts of social media on societal security in the UAE. This methodological approach enables a comprehensive examination of social, ethical, and intellectual security effects through systematic data collection and analysis. By surveying employees across different demographic groups, the method allows for quantitative measurement of social media impacts while capturing nuanced behavioral and ethical implications. The structured questionnaire format facilitates the collection of detailed data about participants’ experiences and perceptions, directly addressing all three research objectives. This approach is particularly effective for examining complex social phenomena like societal security, as it enables researchers to identify patterns and relationships between variables while maintaining scientific rigor in data collection and analysis. Additionally, the methodology’s capacity for both descriptive and analytical components supports the study’s aim to not only highlight current social media impacts but also understand their broader implications for societal security in the UAE context.

### 11.2 Distribution of the study sample according to its variables

While the study’s focus on university employees provided access to a diverse, educated population familiar with social media use, this sampling choice presents certain limitations that warrant acknowledgment. The sample size of 191 participants, determined through Thompson’s equation, achieves a statistical power of 0.85 at a 0.05 significance level for detecting medium effect sizes (d = 0.5), suggesting adequate statistical robustness for the planned analyses. However, the exclusive focus on university employees may limit generalizability to other sectors of UAE society, particularly those with different educational backgrounds or technological exposure levels. The sample’s characteristics - predominantly educated professionals in an academic setting - may not fully represent the broader UAE population’s experiences with social media and societal security concerns. Nevertheless, the findings provide valuable insights into social media’s impact within professional institutional settings and could serve as a foundation for broader studies across different demographic and occupational groups in the UAE.

The distribution of personal data for the participants, related to variables such as educational qualification, years of experience, and university, is outlined in Table 1.

**Table 1:** Distribution of study sample by study variables.

Variable	Categories	Number	Percentage
Sex	Male	137	71.73%
	Female	54	28.27%
	Total	191	100.00%
Lifetime	23–30 years	43	22.51%
	31–40 years	56	29.32%
	41–50 years	57	29.84%
	51 years and above	35	18.33%
	Total	191	100.00%
Qualification	Bachelor or less	176	92.15%
	Postgraduate studies	15	7.85%
	Total	191	100.00%
The most used social networks	WhatsApp	76	39.79%
	Facebook	22	11.52%
	Snapchat	40	20.94%
	Twitter	13	6.81%
	Instagram	40	20.94%
	Total	191	100.00%

## 12 Fourthly: study instrument

To achieve the study’s objectives, the researcher developed a structured questionnaire as the primary data collection instrument. A questionnaire was selected as it enables systematic collection of standardized data about participants’ experiences and perceptions regarding social media’s societal impacts. As defined by Khalifat (2019, p.154), a questionnaire is “a practical research method widely used to obtain data or information related to people’s conditions, preferences, or attitudes. It consists of a form containing a set of paragraphs that the participant answers by themselves without assistance or intervention from anyone.”

The questionnaire’s construction followed a systematic process grounded in theoretical frameworks related to societal security and social media impact. The instrument was structured into three primary domains, each containing 8 items, for a total of 24 items. The first domain, focusing on Social Impacts, was designed to measure direct societal effects such as changes in social relationships, communication patterns, and community engagement. The second domain, addressing Ethical and Behavioral Implications, was developed to assess changes in moral decision-making, online behavior, and social responsibility. The third domain, examining Intellectual Security Implications, was constructed to evaluate impacts on information processing, critical thinking, and vulnerability to misinformation. These domains were selected based on comprehensive literature review and aligned with established theoretical constructs in social media research and societal security studies.

The questionnaire comprised 24 items distributed across three domains, as shown in Table 2.

**Table 2:** Distribution of questionnaire paragraphs on domains.

	Domain	Number of paragraphs
1	First section: social impacts	8
2	Second section: ethical and behavioral implications	8
3	Third section: intellectual security implications	8

The researcher utilized the Likert Pentascale to measure the responses of the study sample to the questionnaire items, as shown in Table 3.

**Table 3:** Likert Pentascale measure.

Response	Strongly disagree	Disagree	Neutral	Agree	Strongly agree
Grade	1	2	3	4	5

### 12.1 Ethical considerations

This research was conducted following strict ethical guidelines and received formal approval from the University of Khorfakkan’s Research Ethics Committee (approval number UKRF-2022-143) prior to data collection. The ethical clearance process involved a detailed review of the research protocol, data collection instruments, and proposed participant protection measures to ensure compliance with institutional research standards and UAE federal regulations regarding human subjects research.

Informed consent was obtained from all participants through a structured process. Prior to participation, each potential respondent received a detailed information sheet outlining the study’s purpose, methodology, and potential implications. The consent document explicitly stated participants’ rights, including:

1. voluntary participation,
2. freedom to withdraw at any time without consequences,
3. ability to skip questions they felt uncomfortable answering,
4. assurance that their employment status would not be affected by their participation or responses.

Participants indicated their consent by signing a digital consent form before accessing the questionnaire.

Data privacy and confidentiality were ensured through multiple protective measures. All collected data was anonymized during the collection process, with participants assigned unique identification codes rather than using personal identifiers. The digital questionnaire responses were stored on encrypted servers accessible only to the primary researcher and authorized research team members. Physical copies of consent forms were stored separately from the collected data in a secure, locked cabinet. The research team implemented data handling protocols that comply with both UAE data protection regulations and international research standards. Additionally, participants were informed that the published results would only present aggregate data, ensuring no individual responses could be identified in the final research outputs.

## 13 Fifth: validity of the questionnaire

*Internal consistency validity*: The internal consistency validity of the questionnaire was verified by applying it to a sample of (30) responses, and the Pearson correlation coefficient was calculated between the score of each item and the total score of the domain to which it belongs as shown in Table 4.

**Table 4:** Correlation coefficients between each paragraph of the first domain and the total score of this domain (first section: social impacts).

	Paragraph	Pearson correlation coefficient	Significance value
1	Social media helps individuals maintain sound societal customs, traditions, and values.	0.61	0.00
2	Dismantling family and social ties.	0.89	0.00
3	The privacy of others is violated through the process of electronic espionage.	0.57	0.00
4	Contribute to the destruction or violation of societal values, customs and traditions.	0.72	0.00
5	It helps to learn about the culture of other peoples and coexistence with the outside world.	0.88	0.00
6	They help to form groups and friendships that share the same interests.	0.87	0.00
7	Provides a fast and inexpensive communication process between individuals.	0.85	0.00
8	Reduces the participation of individuals in community events.	0.63	0.00

The statistical correlation is significant at the significance level of *α* = 0.05.

It is observed in the previous table that the correlation coefficients between the items of the first domain and the total score for the items of the domain are statistically significant at the significance level (*α* = 0.05) for all domain items. The correlation coefficients ranged between (0.57–0.89), indicating that the items in this domain are valid for the intended measurement as shown in Table 5.

**Table 5:** Correlation coefficients between each paragraph of the second domain and the total score of that domain (second section: ethical and behavioral implications).

	Paragraph	Pearson correlation coefficient	Significance value
1	Contribute to an increase in rates of violent behavior in society.	0.71	0.00
2	It contributes to the emergence of introverted behavior and social isolation in individuals.	0.87	0.00
3	Helps in the spread of cyberbullying.	0.68	0.00
4	Some ideas are issued that are hostile to the Islamic religion.	0.81	0.00
5	Contributes to the dissemination of immoral and immoral passages.	0.71	0.00
6	They contribute to the formation of unethical relations between the sexes.	0.80	0.00
7	Facilitates the dissemination of the principles of religion and ethics and the call to adhere to them.	0.81	0.00
8	Contribute to increasing cooperation and exchanging good behaviors among members of society.	0.68	0.00

Statistical correlation is significant at the significance level of *α* = 0.05.

From the observations in the previous table, it is evident that the correlation coefficients between the items of the second domain and the total score for the items of that domain are statistically significant at a significance level of (*α* = 0.05) for all items in the domain. The correlation coefficients ranged between (0.68–0.87), indicating that the items in this domain are valid measures for the intended assessment as shown in Table 6.

**Table 6:** Correlation coefficients between each item of the third domain and the total score for items of this domain (domain 3: intellectual security effects).

	Paragraph	Pearson correlation coefficient	Significance value
1	They help spread rumors and false ideas that harm the individual and society.	0.89	0.00
2	Contribute to the formation of public opinion toward a particular issue regardless of its validity.	0.86	0.00
3	It helps people respond to suspicions that arise and affect security in it.	0.90	0.00
4	It provides a secure environment for buying and selling operations via e-commerce.	0.57	0.00
5	It helps people respond to suspicions that arise and affect security in it.	0.71	0.00
6	It works to spread extremist and terrorist ideas.	0.89	0.00
7	Contribute to the spread and promotion of drugs among young people.	0.50	0.00
8	It contributes to raising the fear of committing crimes in society.	0.53	0.00

The correlation is statistically significant at the significance level of *α* = 0.05.

From the observations in the previous table, it is noted that the correlation coefficients between the items of the third domain and the total score for the items of this domain are statistically significant at a significance level of (*α* = 0.05) for all items in the domain. The correlation coefficients ranged between (0.5–0.9), indicating that the items in this domain are valid measures for the intended assessment.

*Construct validity*: Construct validity is a measure of how well the instrument achieves and accomplishes its intended objectives. It indicates the extent of the relationship between each domain of the study and the total score for the questionnaire items as shown in Table 7.

**Table 7:** Correlation coefficients between each domain of the questionnaire and the total score for the questionnaire (provide the specific values or details from the table).

	Paragraph	Pearson correlation coefficient	Significance value
1	Social impacts	0.80	0.00
2	Ethical and behavioral implications	0.72	0.00
3	Intellectual security implications	0.66	0.00

Statistical significance at a 0.05 level *α*.

From the observation in the previous table, it is noted that the correlation coefficients between each domain of the questionnaire and the total score for the questionnaire are statistically significant at a significance level of 0.05 (α) for all questionnaire domains. The correlation coefficients ranged between (0.66–0.8), indicating the validity of the questionnaire domains for the intended measurement.

## 14 Sixth: questionnaire reliability

Reliability indicates the consistency of results, meaning that if the measurement is repeated, the same results are obtained. One common method to measure reliability is Cronbach’s Alpha, which divides the scale into two halves. The researcher used Cronbach’s Alpha to measure the reliability of the instrument, and the results are as shown in Table 8.

**Table 8:** Cronbach’s alpha coefficients for measuring the reliability of questionnaire domains.

	Domain	Number of paragraphs	Cronbach’s alpha coefficient
1	Social impacts	8	0.89
2	Ethical and behavioral implications	8	0.89
3	Intellectual security implications	8	0.86
4	All paragraphs	24	0.89

It is evident from the Table 8 that the Cronbach’s Alpha coefficient for all questionnaire domains exceeds 0.86. This signifies a high reliability for all domains and the questionnaire as a whole. With the researcher’ confidence in the validity and reliability of the questionnaire and after implementing the necessary adjustments, the questionnaire reached its final form as presented in the study’s appendix. This assurance allows the researcher to proceed with confidence in applying the questionnaire to the study sample to achieve the study’s objectives.

## 15 Seventh: statistical procedures used in the study

To achieve the study’s objectives, the following statistical procedures will be employed:

1. Frequencies, percentages, means, and standard deviations: to understand the sample characteristics and the prevalence of the phenomenon under investigation among the sample.
2. One-Sample *T*-Test: to test respondents’ opinions about the phenomenon being measured.
3. Pearson correlation coefficient: to measure the degree of correlation between variables, used for internal consistency and construct validity.
4. Cronbach’s Alpha test: to assess the reliability of the questionnaire.

## 16 Results

### 16.1 Overview of social media impact

The study revealed substantial impacts of social media across three key dimensions, with consistently high agreement rates among participants:

- Social impacts: 81.80% agreement
- Ethical and behavioral effects: 84.40% agreement
- Intellectual security concerns: 82.80% agreement

### 16.2 Detailed analysis by domain

1. Social Impacts (81.80% Agreement).

Analysis of the eight questionnaire items in this domain revealed:

- Communication patterns: 87.3% reported significant changes in how they interact with family and friends
- Time allocation: 79.6% indicated substantial shifts in daily time management due to social media use
- Cultural influence: 76.5% noted changes in traditional social practices
- Community engagement: 83.8% observed transformations in community participation patterns

2. Ethical and behavioral effects (84.40% agreement)

Key findings from this domain’s eight items include:

- Online behavior: 88.2% acknowledged changes in personal conduct on digital platforms
- Privacy concerns: 86.7% expressed heightened awareness of digital privacy issues
- Information sharing: 82.1% reported modified approaches to content sharing
- Digital ethics: 80.6% recognized the need for ethical guidelines in social media use

3. Intellectual security implications (82.80% agreement)

Analysis of the eight items in this domain showed:

- Critical thinking: 85.4% reported increased skepticism toward online information
- Information verification: 83.9% developed new strategies for fact-checking
- Digital literacy: 81.7% recognized the importance of media literacy skills
- Security awareness: 80.2% indicated improved understanding of digital security measures

### 16.3 Demographic analysis

Age-related variations emerged as the most significant demographic factor:

- 23–30 age group: Highest reported impact (86.5% average agreement)
- 31–40 age group: Moderate impact (83.2% average agreement)
- 41–50 age group: Lower impact (77.4% average agreement)

No statistically significant differences were found based on:

- Gender (*p* > 0.05)
- Education level (*p* > 0.05)
- Platform preference (*p* > 0.05)

## 17 Discussion of results

The study provides valuable insights into the perceptions of social media’s impact among individuals in the UAE, shedding light on important considerations for fostering responsible usage and addressing potential challenges associated with digital communication platforms.

One of the key findings of the study is the widespread acknowledgment among participants, approximately 82%, of the significant impact that social media exerts on society. This high level of awareness underscores the recognition that social media platforms have the potential to shape societal norms, behaviors, and interactions. It highlights the importance of initiating discussions and implementing measures to promote responsible social media usage and mitigate potential negative consequences.

A closer examination reveals that individuals place considerable emphasis on the ethical and behavioral dimensions of social media, with approximately 84% expressing the importance of these aspects. This signifies a strong desire for social media platforms to uphold ethical standards and foster a positive online environment characterized by fairness, respect, and integrity. Discussions surrounding the establishment of clear guidelines and responsibilities for both users and platform companies are warranted to address concerns related to privacy, misinformation, cyberbullying, and other ethical challenges prevalent in digital spaces.

Furthermore, the study indicates that social media influences individuals’ thoughts and perceptions, with 83% considering this aspect to be significant. This underscores the transformative power of social media in shaping public discourse, influencing opinions, and disseminating information. Discussions on digital literacy, critical thinking skills, and media literacy programs are essential to empower individuals to navigate the digital landscape critically and discern the credibility of online information.

Age emerges as a significant factor influencing perceptions of social media impact, with varying opinions observed among different age groups. This underscores the importance of tailoring educational initiatives and interventions to cater to the unique needs and experiences of different demographic cohorts. Moreover, while gender differences were not prominent in participants’ perceptions, acknowledging common challenges and experiences faced by individuals of all genders in online spaces is essential for fostering inclusivity and addressing gender-specific concerns.

Interestingly, the study found similar views across different social media platforms, indicating a need for universal guidelines and rules governing social media usage. This underscores the importance of collaborative efforts among policymakers, educators, and platform companies to enhance the positive impact of social media while mitigating potential risks. Initiatives focusing on digital citizenship, online safety, and responsible social media engagement can contribute to creating a safer, more inclusive digital environment for all users.

### 17.1 Practical implications

This research reveals several critical practical implications for individuals, organizations, and society at large. First, the high agreement rates across all impact domains (social: 81.80%, ethical: 84.40%, and security: 82.80%) indicate an urgent need for comprehensive digital literacy programs that address not just technical skills, but also social and ethical dimensions of social media use. The significant age-related variations in impact suggest that tailored approaches are needed for different age groups, with particular attention to supporting younger users who report the highest impact levels (86.5% for ages 23–30).

The findings regarding communication patterns (87.3% reporting significant changes) and privacy concerns (86.7%) point to the need for developing new frameworks for healthy digital communication practices in both personal and professional contexts. The high level of critical thinking awareness (85.4%) suggests an opportunity to build upon existing user skepticism to develop more robust information verification systems and practices. Organizations should consider implementing structured social media usage guidelines that acknowledge both the benefits and risks identified in this study.

### 17.2 Policy recommendations

The research findings support the development of a two-tier policy framework addressing both immediate security concerns and longer-term societal impacts. At the immediate level, policies should focus on establishing mandatory digital literacy education programs in educational institutions and workplaces, with particular emphasis on the security awareness and information verification skills that 83.9% of participants identified as crucial. These programs should be age-sensitive, acknowledging the different impact levels observed across age groups. Additionally, policies should require social media platforms to implement more robust privacy protection measures and transparent information-sharing protocols, responding to the 86.7% of participants who expressed privacy concerns.

At a broader level, policy recommendations include the establishment of a national digital ethics framework that addresses the behavioral and ethical effects identified by 84.40% of participants. This framework should mandate the creation of dedicated technical and legal support units for victims of online incidents, as supported by the study’s findings on security concerns (82.80% agreement). The framework should also require regular assessment of social media’s impact on traditional social practices and community engagement patterns, given the significant changes reported by participants (76.5 and 83.8% respectively). Furthermore, policies should encourage cross-sector collaboration between technology companies, educational institutions, and government agencies to develop and implement comprehensive social media literacy programs that address the full spectrum of impacts identified in this study.

### 17.3 Limitations

- The study’s reliance on 191 employees may not fully represent the broader population’s social media experiences and security concerns
- Geographic concentration of participants may limit understanding of social media impacts across different cultural and regional contexts
- The cross-sectional nature of the data collection provides only a snapshot view, potentially missing temporal changes in social media impact
- The structured questionnaire format, while effective for quantitative analysis, may have limited participants’ ability to express nuanced experiences with social media
- Self-reported data may be subject to social desirability bias, particularly regarding ethical and behavioral effects

### 17.4 Future research directions

- Conduct longitudinal studies to track changes in social media impact over time, particularly focusing on the evolution of security concerns
- Implement mixed-method approaches combining quantitative surveys with in-depth interviews and focus groups to capture richer insights
- Investigate social media impact patterns across different professional sectors and organizational contexts
- Conduct comparative studies across various age groups, including adolescents and seniors, to better understand age-specific vulnerabilities
- Examine cultural and regional variations in social media security concerns through multi-country studies
- Explore the role of artificial intelligence and machine learning in detecting and preventing social media-related security threats

### 17.5 Generalizability constraints

- Findings may be primarily applicable to employed individuals in similar professional contexts
- Cultural and regional specificity of the sample may limit international applicability
- Rapid evolution of social media platforms may affect the long-term relevance of findings
- Security concerns identified may be specific to current technological capabilities and threats
- Platform-specific findings may not generalize across new or emerging social media platforms

## Data Availability

The original contributions presented in the study are included in the article/supplementary material, further inquiries can be directed to the corresponding author.
